# The Retinoic-Acid-Related Orphan Receptor Alpha May Be Highly Involved in the Regulation of Seasonal Hair Molting

**DOI:** 10.3390/ijms26041579

**Published:** 2025-02-13

**Authors:** Yu Zhang, Xuefei Zhao, Shuqi Li, Suying Bai, Wei Zhang

**Affiliations:** 1 College of Wildlife and Protected Area, Northeast Forestry University, Harbin 150040, China; zhangyunefu@163.com (Y.Z.); zhaoxuefei@nefu.edu.cn (X.Z.); graceli@nefu.edu.cn (S.L.); 2 National Forestry and Grassland Administration Research Center of Engineering Technology for Wildlife Conservation and Utilization, Harbin 150040, China; 3 Detecting Center of Wildlife, State Forestry and Grassland Administration, Harbin 150040, China

**Keywords:** melatonin, *Rorα*, hair follicle stem cell

## Abstract

Seasonal molting in mammals is a crucial survival strategy, yet the underlying molecular mechanisms have not been fully characterized. Melatonin, serving as a bridge for the transmission of photoperiod signals, plays a significant regulatory role in animals’ seasonal molting, and the physiological regulatory effects of melatonin signaling are highly dependent on the retinoic-acid-related orphan receptor alpha (*Rorα*). Hair follicle stem cells (HFSCs) are the most essential cell type in the process of hair follicle regeneration and seasonal replacement. Therefore, this study aims to discuss the regulatory effects of melatonin and its nuclear receptor RORA on HFSCs. This research found that RORA can downregulate cellular proliferation levels by inhibiting the cell cycle of HFSCs, while simultaneously promoting apoptosis in HFSCs and affecting the expression of some genes involved in ferroptosis. RORA can directly bind to the promoter regions of the cyclin genes *Ccna2* and *Ccne1* to regulate their transcription. Melatonin may enhance the viability of HFSCs by downregulating RORA levels. In this study, the impact of melatonin and its nuclear receptor RORA on the viability of HFSCs, along with some of the underlying molecular mechanisms, is characterized. These findings provide a theoretical foundation for research on the regulation of animal hair follicle development.

## 1. Introduction

Hair coat, a unique skin keratinized organ in mammals, serves multiple functions such as temperature regulation, mechanical protection, and secondary sexual characteristics [1,2,3]. It is an important safeguard for animals to adapt to harsh environmental challenges. The hair coat is composed of numerous relatively independent hair follicle structures, and the development of these hair follicles is regulated by the central nervous system and the circulatory system [4]. However, in different locational areas, the development of hair follicles is also influenced by the local microenvironment, demonstrating a relative independence [3]. The development of hair follicles exhibits a distinct rhythmic cycle, which encompasses both an intrinsic developmental cycle and an extrinsic environmental rhythmic cycle. The developmental cycle of hair follicles can be divided into three stages based on morphological characteristics: the anagen phase, the catagen phase, and the telogen phase [5,6]. Throughout the life history of animals, hair follicles repeat this cycle of reconstruction, enabling the replacement of the hair coat. External environmental rhythmicity refers to the influence of seasonal changes on the hair coat development of many mammals. Typically, during the hot summer months, a lighter hair coat aids in heat dissipation, while in the cold winter months, a thicker hair coat helps maintain body temperature. This phenomenon is also known as seasonal molting [7,8].

There is decades of research focused on the developmental mechanisms of the hair coat, particularly the regulatory mechanisms underlying seasonal molting. However, unfortunately, the characterization of the underlying mechanisms for this phenomenon remains insufficient at present. Even so, many factors that influence animal hair coat development have been verified, such as photoperiod, temperature, nutrition, and hormone levels [9,10,11,12,13]. Among these factors, research on the impact of photoperiod on animals’ seasonal molting is relatively advanced. Currently, the academic community generally acknowledges that changes in photoperiod are the most significant drivers of animals’ seasonal molting. Melatonin is an amine hormone synthesized by the pineal gland of vertebrates, exhibiting a wide range of physiological regulatory effects [14,15]. In particular, its role in promoting animal molting has been extensively proven [16,17,18,19]. Interestingly, the skin and hair follicles have been proven to be significant sites for melatonin synthesis outside the pineal gland, suggesting that the development of hair follicles may also depend on the autocrine and paracrine actions of melatonin [20,21]. The synthesis and release levels of melatonin in the central circulatory system are inversely proportional to the duration of light exposure. Additionally, recent research evidence suggests that melatonin may also regulate its own levels through a negative feedback mechanism [22]. Based on melatonin’s sensitivity to photoperiod and its extensive physiological regulatory effects, scholars have proposed a melatonin-mediated photoperiod-seasonal molting regulation model. However, the detailed molecular mechanisms by which photoperiod and melatonin regulate hair coat development remain unclear to date.

A typical hair follicle consists of structures such as the bulb, hair shaft, inner root sheath, arrector pili muscle, sebaceous gland, and connective tissue sheath, which are composed of up to dozens of cell types [23]. These different types of cells work in high coordination to maintain the physiological homeostasis of the hair follicle tissue. Among these cell types, hair follicle stem cells occupy a special position in the development of the hair follicle [24,25,26]. Hair follicle stem cells colonize the bulge area where the arrector pili muscle intersects with the outer root sheath [27]. Upon receiving stimulatory signals, these stem cells rapidly enter an activated state, proliferate swiftly, migrate downwards, and differentiate into progenitor cells, providing the cellular foundation for hair follicle regeneration [28]. Additionally, when the epidermal tissue is damaged, hair follicle stem cells can also migrate upwards to participate in the repair of epidermal tissue injuries [29]. Research has shown that, in addition to possessing the general properties of other adult stem cells, hair follicle stem cells seem to have the ability to perceive their own state. When the degree of their high activation reaches a certain level, hair follicle stem cells will promptly inhibit their own activation and return to a quiescent state to avoid excessive self-consumption [30]. Furthermore, studies have also indicated that the depletion of hair follicle stem cells is the fundamental cause of hair follicle aging [31]. Based on the crucial role of hair follicle stem cells in hair follicle development, it is generally believed that they are the most important cell type in the process of hair follicle regeneration. Studies have been conducted to discuss the regulatory effects of melatonin on the physiological state of hair follicle stem cells, but to date, a more comprehensive characterization of this issue is still needed.

The Eurasian red squirrel (*Sciurus vulgaris*) is a small mammal widely distributed in the high-latitude regions of Europe and Asia [32]. Currently, squirrel farming in China is mainly for ornamental purposes, as pets, and for fur processing, making it an important economic animal. The Eurasian red squirrel exhibits typical seasonal molt characteristics, with molting periods initiating in spring and autumn each year. The morphological differences between its winter and summer fur are significant, and its fur has a wide range of sources, making it an excellent experimental model for studying animal seasonal molt. Therefore, this study aims to use the Eurasian red squirrel as a research model to screen for key functional genes potentially involved in animal seasonal molting. Subsequently, rat hair follicle stem cells are used as a cellular model for functional verification in vitro, with the goal of providing a theoretical basis for research on the regulation of animal seasonal molting.

## 2. Result

### 2.1. RNA-Seq Results of Skin Tissue from Squirrels at Different Stages

**Figure 1:**
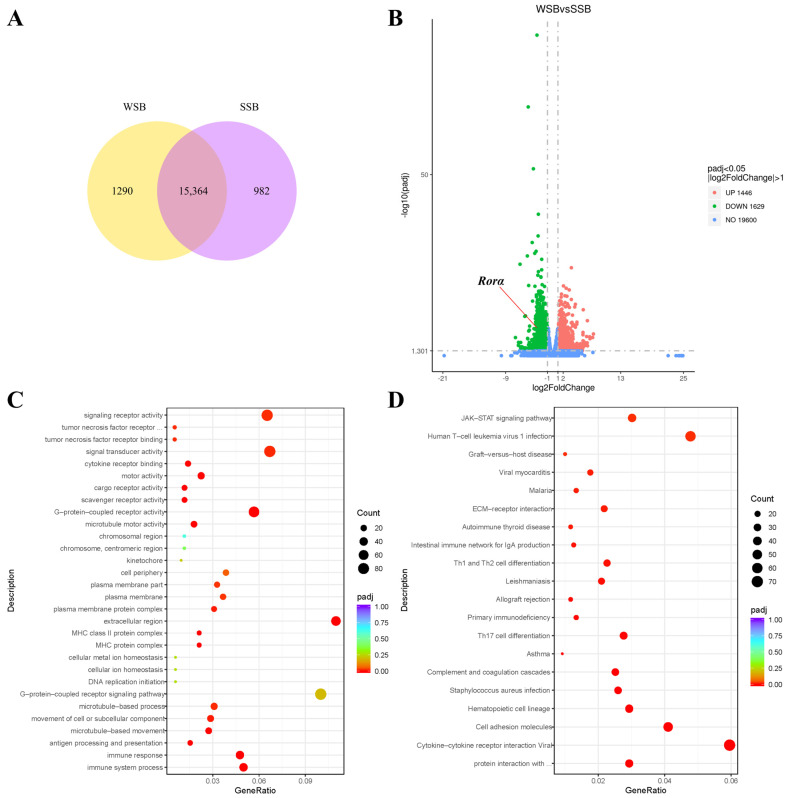
(**A**) The Venn diagram of transcripts from squirrel samples in summer (SSB) and during molting in winter (WSB). (**B**) The Volcano plot of significantly differentially expressed genes in squirrel samples across different seasons, with the *Rorα* gene showing a significant downregulation in transcription levels in samples during the molting period. (**C**) The GO analysis results of significantly differentially expressed genes. (**D**) The KEGG enrichment results of significantly differentially expressed genes.

The RNA-Seq results revealed that 982 unique transcripts were identified in the June samples, while 1290 unique transcripts were identified in the October samples, with 15,364 transcripts common to both (Figure 1A). Compared to summer, a total of 1446 genes were significantly upregulated and 1629 genes were significantly downregulated during the winter molting period (Figure 1B). The GO analysis of the significantly differentially expressed genes showed significant enrichment in terms such as signaling receptor activity, signal transducer activity, G-protein-coupled receptor activity, and extracellular region (Figure 1C). Meanwhile, the KEGG analysis of these genes revealed significant enrichment in pathways such as cell adhesion molecules, human T-cell leukemia virus 1 infection, and cytokine–cytokine receptor interaction (Figure 1D). Among the series of significantly differentially expressed genes, we noted a significant change in the expression level of retinoic-acid-related orphan receptor alpha (*Rorα*). Compared to the summer samples, the transcription level of this gene decreased by nearly 70% during the molting period. As a nuclear receptor for melatonin, the significant decrease in the expression level of this gene suggests significant changes in the physiological regulatory effects mediated by melatonin signaling. Considering the direct regulatory effect of melatonin on animal molting, we believe that *Rorα* may be highly associated with the regulation of the physiological process of seasonal molting in animals.

### 2.2. Activation of RORA Inhibits the Vitality of HFSCs

**Figure 2:**
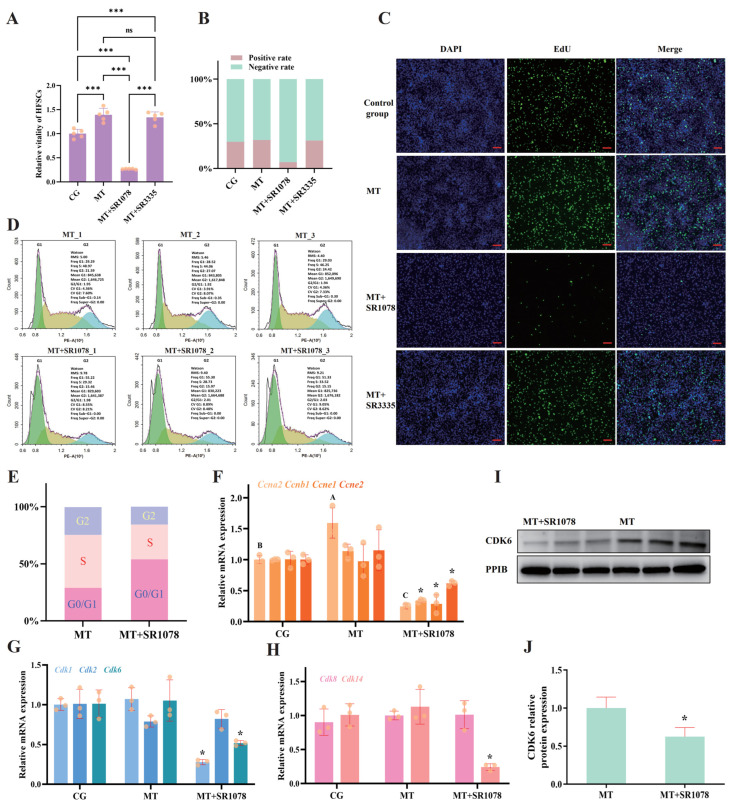
(**A**) Relative viability of HFSCs after treatment with melatonin, SR1078 and SR3335. *** *p* < 0.001. ns, not significant. Subsequent significance markers use the same notation. (**B**) Proportions of Edu-positive HFSCs after treatment with melatonin, SR1078, and SR3335. (**C**) Edu staining to detect cell proliferation after treatment with melatonin, SR1078, and SR3335. The green fluorescent signal represents cells in a proliferating state. Scale bar, 100 μm. (**D**) Flow cytometry analysis of the impact of RORA activation on the cell cycle of HFSCs. (**E**) Proportions of HFSCs in each cell cycle phase under different treatment conditions. (**F**) Relative transcriptional levels of *Ccna2*, *Ccnb1*, *Ccne1*, and *Ccne2* genes in HFSCs after treatment with melatonin and SR1078. The absence of the same letter between different treatment groups for the same gene indicates that the difference is statistically significant, with a *p*-value of less than 0.05. The asterisk (*) on the bar chart indicates that the gene has significant differences from all other groups, with a *p*-value less than 0.05 but greater than 0.01. Subsequent significant markers use the same notation. (**G**) Relative transcriptional levels of *Cdk1*, *Cdk2*, and *Cdk6* genes in HFSCs after treatment with melatonin and SR1078. The absence of significance symbols indicates that there is no statistical significance between the groups. Subsequent significance markers use the same notation. (**H**) Relative transcriptional levels of *Cdk8* and *Cdk14* genes in HFSCs after treatment with melatonin and SR1078. (**I**) WB detection of the effect of RORA activation on the CDK6 protein in HFSCs. (**J**) Relative expression of CDK6 in HFSCs after SR1078 treatment.

To validate our hypotheses regarding the physiological effects of *Rorα*, we treated primary rat hair follicle stem cells with melatonin, as well as with an additional RORA agonist (SR1078) and antagonist (SR3335), and assessed cell viability levels using the CCK-8 assay (Figure 2A). The experimental results showed that the cell count in the melatonin-only treatment group was nearly 50% higher than that in the control group, indicating that melatonin significantly upregulates the vitality of HFSCs, which aligns with our traditional understanding. However, the cell count in the combined melatonin and SR1078 treatment group was only about 25% of that in the control group, suggesting that RORA activation induced by SR1078 significantly reversed the upregulation of HFSC vitality by melatonin, indicating that RORA itself has a significant inhibitory effect on HFSC vitality. There was no significant difference in cell viability between the combined melatonin and SR3335 treatment group and the melatonin-only treatment group, but both showed a significant increase in cell viability compared to the blank control group. Since changes in both cell proliferation and cell death levels can lead to significant alterations in cell viability, we first analyzed changes in cell proliferation levels. The Edu assay results revealed that melatonin treatment enhanced HFSC proliferation, and there was no significant difference between the combined melatonin and SR3335 treatment and the melatonin-only treatment, which is consistent with the CCK-8 cell viability assay results. However, the combined melatonin and SR1078 treatment again reversed the promoting effect of melatonin on cell proliferation, indicating that RORA activation in HFSCs induced by SR1078 significantly inhibits cell proliferation, which may be the key reason for its significant inhibition of cell viability (Figure 2B,C).

### 2.3. The Impact of RORA Activation on Cell Cycle of HFSCs

After confirming the inhibitory effects of RORA activation on cell viability and proliferation in HFSCs, we first examined its impact on the cell cycle of HFSCs using flow cytometry. The results showed that, compared to melatonin treatment alone, additional RORA activation induced by SR1078 caused a significant increase in the proportion of cells in the G0/G1 phase and an upregulation of the ratio of G0/G1 phase cells to S phase cells. An elevated ratio is indicative of cell cycle arrest. Therefore, we believe that the arrest of HFSCs in the G0/G1 phase by RORA is the main reason for inhibiting cell proliferation (Figure 2D,E). Having reached this conclusion, we attempted to analyze the changes in the levels of a series of cyclins and cyclin-dependent kinases (CDKs) involved in the cell cycle. qPCR results showed that only the mRNA level of cyclin *Ccna2* was increased by melatonin treatment, while the mRNA levels of *Ccnb1*, *Ccne1*, and *Ccne2*, although changed after melatonin treatment, did not show significant differences compared to the control group. Notably, after additional treatment with SR1078, the transcription levels of these cyclin genes all decreased significantly, with *Ccna2*, *Ccnb1*, and *Ccne1* transcription levels being only about 25% of those in the blank control group and the *Ccne2* transcription level being only 70% of that in the blank control group (Figure 2F). The qPCR results for a series of CDKs were highly similar to those for cyclins. Melatonin treatment affected the levels of *Cdk1*, *Cdk2*, *Cdk6*, *Cdk8*, and *Cdk14*, but the differences compared to the control group were not significant. However, additional SR1078 treatment significantly downregulated the mRNA levels of *Cdk1*, *Cdk6*, and *Cdk14* but did not affect the transcription levels of *Cdk2* and *Cdk8* (Figure 2G,H). Since the cell cycle analysis showed that RORA activation causes G0/G1 phase arrest in HFSCs, and the *Cdk6* gene is highly correlated with the transition from the G0/G1 phase to the S phase, we further verified changes in its protein level through WB. The results showed that the CDK6 protein level was also decreased by SR1078 treatment (Figure 2I,J). Based on these results, we believe that the inhibitory effects of RORA activation on cyclins and CDKs may be the key reasons for its impact on the HFSC cell cycle, ultimately leading to decreased cell proliferation and viability in HFSCs.

### 2.4. RORA Activation Upregulates Apoptosis in HFSCs

**Figure 3:**
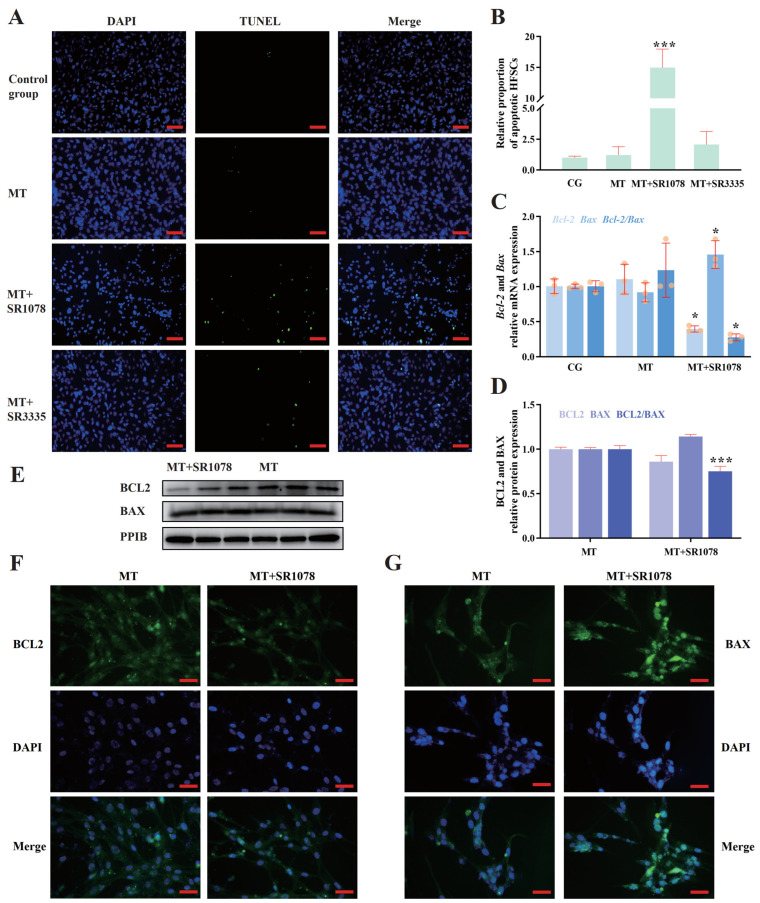
(**A**) Detection of apoptosis in HFSCs after different treatments using the TUNEL method. The green fluorescent signal indicates apoptotic cells. Scale bar, 50 μm. (**B**) Relative proportions of apoptotic cells after different drug treatments. (**C**) Relative transcriptional levels of *Bcl2* and *Bax* genes in HFSCs after treatment with melatonin and SR1078. (**D**) Relative expression of BCL2 and BAX in HFSCs after SR1078 treatment. (**E**) WB detection of the effects of RORA activation on BCL2 and BAX protein expression in HFSCs. (**F**) The immunofluorescence detection result for the BCL2 protein. Scale bar, 25 μm. (**G**) The immunofluorescence detection result for the BAX protein. Scale bar, 25 μm.

Cell death levels are another crucial factor influencing cell viability, so we first discuss the level of apoptosis in HFSCs. The TUNEL assay results indicate that there are no significant differences in the levels of apoptosis among the groups treated with melatonin alone, melatonin combined with SR3335, and the blank control group, all maintaining relatively low levels of apoptosis. However, the combination of melatonin and SR1078 results in a significant increase in the level of apoptosis (Figure 3A,B). Therefore, we believe that the upregulation of apoptosis in HFSCs by RORA activation may be another important reason for its impact on cell viability. The *Bcl2* gene family is highly related to the mitochondrial apoptosis pathway, and a decrease in the ratio of *Bcl2* to *Bax* gene expression levels is considered one of the important indicators of apoptosis. Consistent with the TUNEL assay results, qPCR results show that the combined treatment of HFSCs with melatonin and SR1078 results in a significant downregulation of *Bcl2* mRNA levels and a significant upregulation of *Bax* transcription levels, leading to a significant decrease in the ratio of their transcription levels (Figure 3C). The WB assay results show that the trends in BCL2 and BAX protein levels are consistent with the qPCR results, but the differences are not significant (Figure 3D,E). This may be due to the lag in protein level changes, but it is noteworthy that the ratio of their protein levels has decreased significantly. The immunofluorescence assay results for BCL2 and BAX proteins are shown in Figure 3F,G.

### 2.5. Detection of Indicators Related to Cellular Ferroptosis

**Figure 4:**
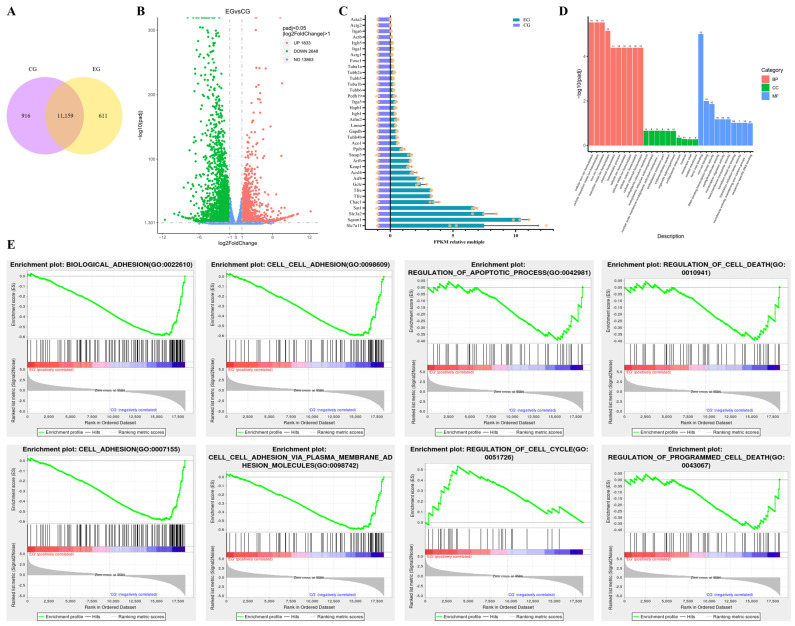
(**A**) The Venn diagram of transcriptome profiles in HFSCs treated with melatonin alone (CG) versus combined treatment with melatonin and SR1078 (EG). (**B**) The Volcano plot of differentially expressed genes after treating HFSCs with melatonin alone versus a combination of melatonin and SR1078. (**C**) Relative FPKM of selected differentially expressed genes. (**D**) The GO enrichment results of highly expressed genes in HFSCs treated with a combination of melatonin and SR1078 compared to melatonin alone. (**E**) Partial results of the GSEA.

After confirming that RORA has an apoptotic effect on HFSCs, we attempted to analyze whether RORA has other pathways that upregulate the level of HFSC death. Therefore, we performed an RNA-Seq analysis (*n* = 3) on cells co-treated with melatonin and SR1078 (experimental group) and used HFSCs treated solely with melatonin as the control group. The Pearson correlation coefficient between biological replicates was greater than 0.92, indicating good reproducibility within the groups (Figure S1). A total of 11,159 co-expressed transcripts were identified between the groups, with 916 unique transcripts in the control group and 611 unique transcripts in the experimental group (Figure 4A). A total of 4481 significantly differentially expressed genes were identified, with 1833 upregulated and 2648 downregulated transcripts in the experimental group compared with the control group (Figure 4B). We exhibited the relative changes in FPKM (fragments per kilobase million) values for some of the genes (Figure 4C). The results of clustering of significantly differentially expressed transcripts are presented in Figure S2, and a list of partial differentially expressed genes is provided in Table S1. The GO analysis of the significantly upregulated genes revealed the significant enrichment of multiple terms related to cellular iron ion homeostasis, ferric iron binding, and other associated processes (Figure 4D). The KEGG analysis indicated the significant enrichment of pathways, including pathways in cancer (Figures S3–S5). According to the gene set enrichment analysis (GSEA), various aspects of functionality, including biological adhesion, cell adhesion, cell–cell adhesion, cell–cell adhesion via plasma membrane adhesion molecules, regulation of apoptotic process, regulation of the cell cycle, regulation of cell death, and regulation of programmed cell death, were specifically enriched in HFSCs (Figure 4E).

**Figure 5:**
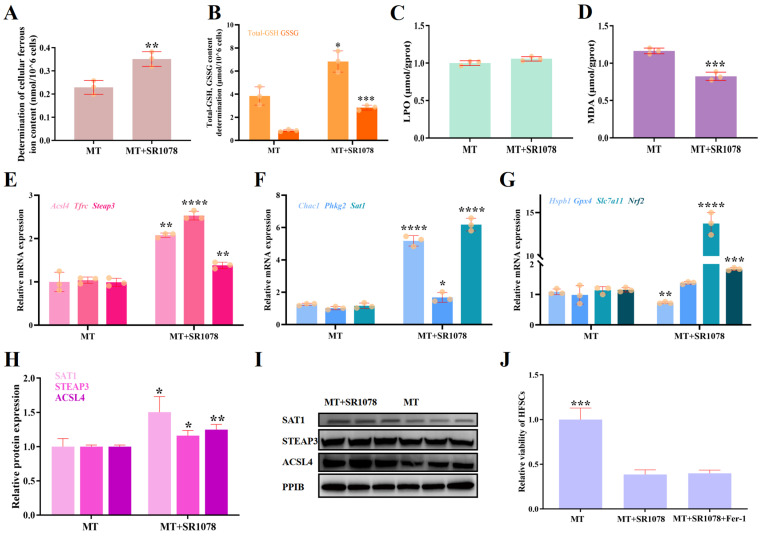
(**A**) Activation of RORA enhances the ferrous ion content of HFSCs. ** 0.001 < *p* < 0.01. Subsequent significance markers use the same notation. (**B**–**D**) Effects of RORA activation on total glutathione and oxidized glutathione (**B**), lipid peroxide (LPO) (**C**), and malondialdehyde (MDA) (**D**) in HFSCs. (**E**) Relative transcriptional levels of *Acsl4*, *Tfrc*, and *Steap3* genes in HFSCs after treatment with melatonin and SR1078. **** *p* < 0.0001. (**F**) Relative transcriptional levels of *Chac1*, *Phkg2*, and *Sat1* genes in HFSCs after treatment with melatonin and SR1078. (**G**) Relative transcriptional levels of *Hspb1*, *Gpx4*, *Slc7a11*, and *Nrf2* genes in HFSCs after treatment with melatonin and SR1078. (**H**) Relative protein expression of SAT1, STEAP3, and ACSL4 in HFSCs. (**I**) WB detection of the effects of RORA activation on SAT1, STEAP3, and ACSL4 protein levels in HFSCs. (**J**) Ferroptosis inhibitor Fer-1 did not alter the suppression of HFSC viability caused by RORA activation.

In the transcriptome sequencing results, we observed the significant differential expression of numerous genes involved in cellular ferroptosis (Table S1). This led us to hypothesize that the activation of RORA might regulate cell viability by affecting the level of ferroptosis. To investigate this, we measured several biochemical indicators related to ferroptosis in the cells. The results showed that compared to the treatment with melatonin alone, the combination of SR1078 and melatonin led to an approximately 1.5-fold increase in the level of ferrous ions in the cells (Figure 5A). However, the contents of total glutathione (total-GSH) and oxidized glutathione (GSSG) also increased significantly with the addition of SR1078 (Figure 5B). Unrestricted peroxidation of lipids is a crucial indicator of cellular ferroptosis. Our measurements revealed that SR1078 had almost no effect on the level of lipid peroxide (LPO) in HFSCs (Figure 5C). Malondialdehyde (MDA), an important end product of lipid peroxidation reactions, is another crucial marker for evaluating cellular ferroptosis. Notably, the amount of MDA decreased significantly with the intervention of SR1078 (Figure 5D).

After measuring the biochemical indicators related to ferroptosis in cells, we further examined the expression levels of genes that positively regulate ferroptosis (*Acsl4*, *Tfrc*, *Steap3*, *Chac1*, *Phkg2*, and *Sat1*) and those that negatively regulate it (*Hspb1*, *Gpx4*, *Slc7a11*, and *Nrf2*) using qPCR and WB analysis (Figure 5E–I). The qPCR results showed an increase in the transcription levels of genes that positively regulate ferroptosis, including *Acsl4*, *Tfrc*, *Steap3*, *Chac1*, *Phkg2*, and *Sat1*. Notably, the elevation in the expression of *Tfrc* and *Steap3* was consistent with the increase in ferrous ion content observed in the biochemical assays, suggesting that these changes in gene expression may be significant contributors to the elevation of ferrous ions in the cells. Among the genes that negatively regulate ferroptosis, the transcription level of *Hspb1* decreased, while the transcription level of Gpx4 increased. Notably, the transcription levels of *Slc7a11* and *Nrf2* showed significant increases. It is worth mentioning that the changes in the *Slc7a11* and *Gpx4* transcription levels are likely a key factor responsible for the significant increases in total-GSH and GSSG observed after treatment with SR1078.

Through the detection of ferroptosis indicators in cells, we observed opposing trends in the changes of factors that promote and those that inhibit ferroptosis. As a result, it is not straightforward to directly determine the impact of RORA activation on the level of ferroptosis in HFSCs. Therefore, we attempted to employ Ferrostatin-1, an inhibitor of ferroptosis, to treat HFSCs. By analyzing whether Ferrostatin-1 could rescue the inhibition of HFSC viability caused by SR1078, we aimed to gain insights into the function of RORA. However, the experimental results showed that the addition of Ferrostatin-1 did not significantly affect cell viability and failed to rescue the inhibition of HFSC viability caused by RORA activation (Figure 5J).

Based on the comprehensive experimental results, we tend to believe that the activation of RORA does not enhance the level of ferroptosis in HFSCs. Instead, it may directly or indirectly influence the expression levels of certain genes involved in the cellular ferroptosis process, leading to changes in biochemical indicators related to ferroptosis, such as the content of ferrous ions. However, these biochemical changes are not uncontrollable, and cells can promptly reverse the ferroptosis tendency through regulatory mechanisms like increasing GSH intake.

### 2.6. Analysis of the Interaction Between RORA and Target Genes

**Figure 6:**
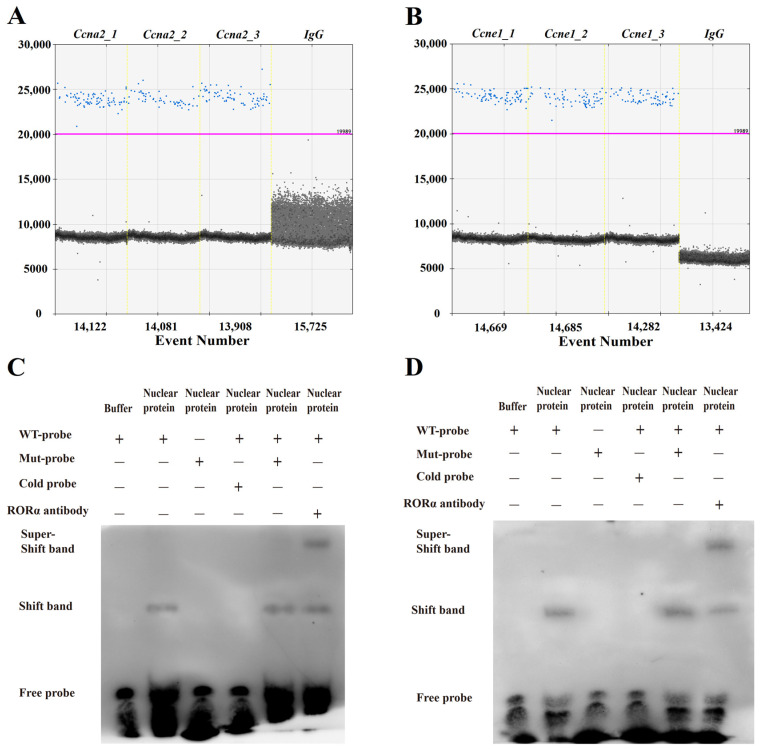
(**A**) CUT&RUN, coupled with droplet digital PCR, was employed to examine the binding interaction within RORA and the promoter regions of *Ccna2*. Blue dots denote positive droplets, while gray dots signify negative droplets, with the horizontal axis depicting the total number of droplets analyzed. (**B**) CUT&RUN, coupled with droplet digital PCR, was employed to examine the binding interaction within RORA and the promoter regions of *Ccne1*. Blue dots denote positive droplets, while gray dots signify negative droplets, with the horizontal axis depicting the total number of droplets analyzed. (**C**) The result of the EMSA for detecting the binding relationship between RORA and the *Ccna2* promoter region. (**D**) The result of the EMSA for detecting the binding relationship between RORA and the *Ccne1* promoter region.

As described in the aforementioned research findings, the activation of RORA regulates various physiological effects in HFSCs and influences the expression levels of a series of genes. However, it remains unclear whether RORA’s regulation of these gene expressions is direct or indirect. Considering the inhibitory effect of RORA activation on the cell cycle of HFSCs and its strong suppression of cyclin expression levels, we validated the interaction between RORA and the cyclin genes *Ccna2* and *Ccne1*. Firstly, we enriched DNA fragments of RORA’s downstream target binding sites using CUT&RUN and detected the presence of DNA fragments from the promoter regions of *Ccna2* and *Ccne1* genes in the enriched products using ddPCR (*n* = 3). Additionally, we set up a control group using only IgG antibodies to avoid false-positive results. The experimental results showed that the detection of the promoter regions of the *Ccna2* and *Ccne1* genes in the enriched products was positive, while the IgG control group showed negative results (Figure 6A,B). These findings indicate that RORA can bind to the promoter regions of these genes and may thereby participate in the regulation of their transcription levels. To further confirm whether the binding of RORA to the *Ccna2* and *Ccne1* genes is direct or depends on other proteins, we conducted verification using EMSA. The EMSA results showed that both the wild-type probes of the *Ccna2* and *Ccne1* promoter regions could form nucleic acid–protein complexes when incubated with nuclear proteins, resulting in the production of shifted bands. Additionally, excess mutant probes could not prevent the formation of wild-type probe–protein complexes, demonstrating no effect on the production of shifted bands. However, excess cold probes could competitively inhibit the formation of wild-type probe–protein complexes, leading to the disappearance of shifted bands. Most importantly, when a specific antibody against the RORA protein was added to the system, the nucleic acid–protein complex underwent a super-shift due to the further increase in molecular weight, producing a super-shift band (Figure 6C,D). These results indicate that the RORA protein can directly bind to the promoter regions of the *Ccna2* and *Ccne1* genes to regulate their expression.

## 3. Discussion

Research focusing on the regulation of animal hair coat development can not only deepen our understanding of animal adaptive evolution but also provide a theoretical foundation for the development of industries such as wool processing. The Eurasian red squirrel exhibits typical seasonal molting characteristics and possesses the advantages of being small in size and widely available. More specifically, China’s squirrel breeding industry is currently at a new development stage, with industries such as pet squirrel breeding for ornamental purposes and squirrel fur accessories entering a rapid development phase. Therefore, this study adopts the Eurasian red squirrel as an animal model to analyze the potential mechanisms underlying seasonal molting in animals.

Based on RNA-Seq results, we hypothesize that the *Rorα* gene may be highly related to seasonal molting in animals. Therefore, we discuss the regulatory role of this gene on the physiological state of hair follicle stem cells. Research has shown that the *Rorα* gene is highly correlated with biological rhythms. *Rorα* and *Rev-Erbα* are considered important components of the circadian clock system [33,34,35,36,37]. Considering that animal hair follicle development and seasonal molting also fall within the category of biological rhythmic phenomena and that studies on goats have demonstrated the potential involvement of *Rorα* in regulating hair follicle development, these are important reasons for us to select *Rorα* as the target gene for our research [38,39]. When the *Rorα* gene was first discovered, it was classified as an orphan receptor, meaning its ligand was unknown [35]. However, as subsequent research progressed, some scholars proposed that melatonin is the natural ligand of RORA, a view that is still accepted by the academic community [40,41,42]. In recent years, however, some studies have presented contrary viewpoints, with many scholars pointing out that melatonin and RORA do not seem to have binding ability [43]. But what is clear now is that melatonin has an impact on the expression level of *Rorα*, and our preliminary research results also indicate that melatonin has a significant inhibitory effect on *Rorα* expression [44,45,46]. More importantly, we found that melatonin membrane receptors are not expressed in rat hair follicle stem cells, only *Rorα* is expressed, which further highlights the importance of *Rorα* in hair follicle development. Considering the regulatory role of melatonin in hair follicle development and seasonal molting in animals, this is another key factor in our choice to focus on *Rorα* in this study [47,48,49].

In the results of cellular proliferation level tests, we found that SR1078 treatment leads to cell cycle arrest and a significant decrease in cellular proliferation levels. We speculate that the regulation of HFSCs’ proliferation levels by melatonin is highly correlated with its inhibitory effect on RORA expression. Additionally, we analyzed how RORA regulates HFSC death. In this section, we focused on two forms of programmed cell death: apoptosis and ferroptosis. Through experiments such as TUNEL, we confirmed the apoptosis-promoting role of RORA, a conclusion that aligns with other related studies [50,51]. However, whether RORA can affect the viability of HFSCs through ferroptosis still requires further confirmation. Based on the results of this study, we believe that RORA regulates the expression levels of some genes involved in the ferroptosis process, leading to an increase in cellular ferrous ion levels and a tendency to promote ferroptosis. However, these changes are promptly responded to by the cells, which reduce the levels of intracellular lipid peroxides by increasing glutathione intake, thereby reversing the trend of ferroptosis. It should be noted that the conclusions of this study are entirely based on observations from live cells and may be subject to survivorship bias; thus, more evidence is needed to fully characterize this issue. In related research, melatonin has been found to inhibit apoptosis in various cell types of animal hair follicles [48,52]. Combining these findings with the results of our study, we speculate that melatonin’s anti-apoptotic effect may be highly correlated with RORA. Additionally, in this study, melatonin treatment alone did not show a significant anti-apoptotic effect compared to the blank control, which may be due to the already low level of apoptosis in the blank control group, as evidenced by the TUNEL staining results.

In this research, we also focused on the molecular mechanism by which RORA regulates the expression of related genes. Using techniques such as CUT&RUN and EMSA, we discovered that RORA can directly bind to the promoter regions of the *Ccna2* and *Ccne1* genes, suggesting that RORA, as a nuclear receptor, can influence the transcription levels of these genes through direct binding. Additionally, literature reports indicate that RORA not only has the ability to directly regulate gene transcription but can also modulate downstream gene transcription as a transcriptional cofactor [53]. Furthermore, RORA can regulate the levels of other transcription factors to indirectly regulate downstream target genes. These diverse forms of gene regulation imply the existence of a complex regulatory network behind RORA’s modulation of cellular states. Understanding the detailed molecular mechanisms behind this could deepen our comprehension of the regulation of animal hair follicle development. Considering the reverse regulatory effect of melatonin on *Rorα* levels and the impact of melatonin and *Rorα* on HFSC viability, we speculate that the shortened photoperiod during the winter molting season leads to an increase in melatonin levels in animals. High levels of melatonin then inhibit *Rorα* levels, which in turn promotes HFSC viability, facilitating the transition from summer to winter coats in animals. Research and applications of melatonin in the field of animal fur products have been ongoing for decades. However, due to the complex physiological effects and regulatory mechanisms of melatonin, we are still far from fully characterizing its effects. Based on the results of this study, we believe that the *Rorα* gene is a promising starting point for investigating the physiological regulatory effects of melatonin. Additionally, the *Rorα* gene holds potential as a new target in research and industrial applications related to animal coat development.

## 4. Methods

### 4.1. Cellular Model and Drug Treatment

In October, prior to the visually observable initiation of the molting period, and in June during the summer hair coat, three 1-year-old male Eurasian red squirrels that were outdoor-bred were purchased from the Wuying Squirrel Farm in Yichun City, Heilongjiang Province, China. The experimental animals were then transferred to the laboratory for outdoor breeding and fasted for 48 h, during which they had free access to water. Following euthanasia by CO_2_ asphyxiation, skin tissue with intact hair follicles was collected from their backs and rapidly transferred to liquid nitrogen for freezing and subsequent transcriptome sequencing. The cellular model used in this study was primary rat hair follicle stem cells, obtained using a method referenced from studies by Oshima, Rochat, and Shwartz et al. [54,55,56]. The brief procedure for obtaining these cells is as follows: After euthanasia by CO_2_ asphyxiation, the snout tissue of 8-week-old male Sprague Dawley rats was isolated and submerged in 75% ethanol for surface sterilization. Using micro-neural forceps and a 1 mL syringe needle under a dissecting microscope, the medial dermal tissue was stripped away to expose the hair follicle connective tissue sheath. After complete isolation of the hair follicles, the connective tissue sheath was cut open, and tissues such as the hair bulb, hair shaft, and arrector pili muscle were resected to obtain the hair follicle bulge region. Primary culture was then performed using the tissue explant method under conditions of 37 °C and 5% CO_2_. The obtained cells were analyzed for the expression of hair follicle stem cell markers by droplet digital PCR and immunofluorescence. The medium used in this study was Dulbecco’s Modified Eagle Medium/Nutrient Mixture F-12 (DMEM/F12) containing 2% fetal bovine serum (Gibco, London, UK, A5256701), supplemented with GlutaMAX™ (Gibco, 35050061) and final concentrations of 50 ng/mL EGF (MCE, HY-P7092) and bFGF (MCE, Monmouth Junction, NJ, USA, HY-P5321). The final concentration of melatonin used in the experimental groups was 1000 ng/L, while SR1078 (RORA agonist) and SR3335 (RORA inhibitor) were used at concentrations of 10 μM. The drug treatment duration for the cellular model was 24 h.

### 4.2. RNA-Seq

The library construction and sequencing processes for both the Eurasian red squirrel skin tissue samples and the primary rat hair follicle stem cell samples, based on the Illumina high-throughput sequencing platform, were entrusted to Beijing Novogene Bioinformatics Technology Co., Ltd. (Beijing, China). A brief overview of the analysis workflow is as follows: A NEBNext^®^ Ultra™ RNA Library Prep Kit for Illumina^®^ (NEB, E7530) was used for library construction, and the qualified sequencing libraries were subjected to high-throughput sequencing using the Illumina sequencing platform. The raw results were converted into fastq-formatted Raw Reads through CASAVA base calling and further processed to remove sequencing adapters and low-quality Reads, resulting in Clean Reads for subsequent analysis. The HISAT2 (v2.2.0) software was used to align the Clean Reads to the reference genome, obtaining the location information of Reads on the reference genome. The expression level of transcripts was described by the FPKM value, and a differential expression analysis was performed using DESeq2. Significant differentially expressed genes were determined based on the criteria of padj ≤ 0.05 and log2.Fold_change ≥ 1. Subsequently, KEGG and GO analyses were conducted. GSEA was executed utilizing the GSEA (v4.0.2) algorithm in conjunction with MSigDB (v7.0), employing the default settings.

### 4.3. RNA Isolation and Real-Time qPCR

The purification of total RNA and the synthesis of cDNA for the samples were performed using a Thermo Scientific GeneJET RNA Kit (Thermo Fisher, Waltham, MA, USA, K0732) and a PrimeScript™ RT reagent Kit with gDNA Eraser (Takara, Kusatsu, Japan, RR047A), respectively, following the instructions provided in the kits. Real-time qPCR was conducted using SsoAdvanced™ Universal SYBR^®^ Green (Bio-Rad, Hercules, CA, USA, 1725270), with the reaction system and program set according to the manufacturer’s instructions. The Bio-Rad CFX384 Real-Time PCR Detection System was used for qPCR detection, and the default program of the instrument was employed for melting curve analysis. The relative expression levels of genes were calculated using the 2^−ΔΔCt^ method. The information on all primers used in this study is provided in the Supplementary Materials.

### 4.4. Protein Isolation and Western Blotting

Cells were subjected to a routine digestion process and centrifuged to obtain a precipitate. RIPA (Radioimmunoprecipitation assay buffer) containing a final concentration of 1 mmol/L phenylmethanesulfonyl fluoride was added, and the cells were repeatedly pipetted to mix evenly. The mixture was then vortexed at maximum speed using a vortex mixer and left to stand on ice for 10 min, with vortexing repeated several times during this period to ensure complete cell lysis. After centrifugation, the supernatant, which contained the total cellular protein, was collected. Protein concentration was determined using the BCA method (Solarbio, Beijing, China, PC0020), and the sample volume was adjusted before adding 4× Protein SDS-PAGE Loading Buffer (Takara, 9173). The protein samples were then denatured by heating at 99 °C for 10 min. SDS-PAGE gels with gradients of 4–12% and 4–20% (Genscript, Piscataway, NJ, USA, M00653; M00656) were used to separate the protein bands according to the molecular weight of the target proteins. Methanol-activated PVDF membranes were used for protein blotting at a condition of 200 mA for 1 h. After blotting, the membranes were blocked with 5% BSA (in TBST) for 1 h. Following blocking, the membranes were incubated with the primary antibody overnight at 4 °C. The next day, the primary antibody was removed, and the membranes were washed three times with TBST buffer for 10 min each. The membranes were then incubated with the secondary antibody at room temperature for 1 h, followed by another series of washes with TBST buffer. ECL chemiluminescent solution and the Bio-Rad ChemiDoc MP Imaging System were used for image acquisition, and ImageJ software was employed for gray-scale analysis of the protein bands. Information on the antibodies used in this study is provided in the Supplementary Materials.

### 4.5. Immunofluorescence

Cell slides were prepared and equilibrated for 24 h. After drug treatment for 24 h, the liquid was removed, and the slides were rinsed several times with PBS buffer to remove residual medium and cellular debris. The cells were then fixed with acetone for 15 min and permeabilized with 0.5% Triton X-100 at room temperature for 20 min. After removing the liquid and rinsing again, the slides were blocked with goat serum at room temperature for 30 min. Following blocking, the primary antibody was incubated overnight at 4 °C. The next day, the primary antibody was removed, and the slides were washed multiple times with PBS buffer. The secondary antibody was then added and incubated at room temperature for 1 h. After thoroughly washing the slides with PBS to remove any residual antibody, they were mounted with an anti-fluorescence quenching mounting medium containing DAPI and transferred to a fluorescence microscope for observation.

### 4.6. Cell Viability

Cells were seeded at a density of 3000 cells per well in a 96-well cell culture plate and pre-cultured for 24 h. The cells were then treated with melatonin alone, melatonin combined with SR1078, and melatonin combined with SR3335, and a blank control group was set up with only DMSO treatment. After 24 h of drug treatment, CCK-8 (Cell Counting Kit-8) reagent (Beyotime, Jaingsu, China, C0037) was added to each well, and the plate was returned to the incubator for an additional 1 h of culture. Following the incubation, the absorbance was measured at 450 nm using a Multiskan™ FC Microplate Reader (Thermo Fisher, 51119180ET).

### 4.7. Cell Proliferation

Cells were seeded in a 6-well culture plate and pre-cultured for 24 h to stabilize their state. The experimental grouping was the same as that for cell viability detection. After 24 h of drug treatment, a final concentration of 10 μM of Edu (5-Ethynyl-2′-Deoxyuridine) working solution (Beyotime, C0071S) was added, and the cells were incubated for an additional 2 h. The cell culture medium was then removed, and the cells were fixed with 4% paraformaldehyde for 15 min. The fixative was removed, and the cells were washed three times with PBS buffer for 5 min each. Next, 0.3% Triton X-100 was added to permeabilize the cells at room temperature for 10 min, followed by another wash. Click reaction solution was added, and the cells were incubated at room temperature in the dark for 30 min. The solution was then removed, and the cells were washed three times again with PBS buffer. The cells were mounted with an anti-fluorescence quenching mounting medium containing DAPI and transferred to a fluorescence microscope for observation.

### 4.8. Flow Cytometry Analysis

Cells seeded in a 6-well culture plate were pre-cultured for 24 h and then treated with melatonin alone or melatonin combined with SR1078 for another 24 h. The cells were then collected through a routine digestion process to obtain a cell precipitate and fixed with pre-cooled 70% ethanol overnight. Cell cycle analysis was performed using an Agilent NovoCyte flow cytometer (Santa Clara, CA, USA) and an FxCycle™ PI/RNase kit (Invitrogen, F10797, Carlsbad, CA, USA). The procedures were followed according to the instructions provided with the kit.

### 4.9. TUNEL

Cells were treated according to the experimental grouping for cell viability detection. After removing the cell culture medium, the cells were washed with PBS buffer. They were then fixed with 4% paraformaldehyde for 30 min and washed again to ensure complete removal of the fixative. Next, 0.3% Triton X-100 (in PBS) was added to permeabilize the cells at room temperature for 5 min. One-step TUNEL cell apoptosis detection was performed using a Fluorescein (FITC) Tunel Cell Apoptosis Detection Kit (Servicebio, Wuhan, China, G1501) following the instructions provided with the kit. The level of cell apoptosis was observed under a fluorescence microscope.

### 4.10. Measurement of Cell Ferroptosis-Related Indicators

Levels of ferrous ions (Elabscience, Houston, TX, USA, E-BC-K881-M), total glutathione and oxidized glutathione (Elabscience, E-BC-K097-M), malondialdehyde (Elabscience, E-BC-K025-M), and lipid peroxide (Elabscience, E-BC-K176-M) were measured in each group of cells, following the manufacturer’s instructions. For the ferrostatin-1 rescue experiment, HFSCs were seeded in 96-well plates and allowed to equilibrate for 24 h before drug treatment. Cell viability was then assayed using the CCK-8 method.

### 4.11. Cleavage Under Targets and Release Using Nuclease (CUT&RUN)

After obtaining cell precipitates through routine digestion, CUT&RUN was performed using a CUT&RUN Assay Kit (Cell Signaling Technology, Danvers MA, USA, #86652) and DNA Purification Buffers and Spin Columns (Cell Signaling Technology, #14209) to enrich DNA fragments bound to the downstream target gene binding sites of RORA. The reaction system and experimental procedures were followed according to the instructions provided with the kit. Additionally, a control group was set up using only IgG antibodies. Primers targeting the promoter region of the gene of interest were designed for droplet digital PCR amplification. Positive results indicated the presence of binding between RORA and the promoter region of the gene of interest.

### 4.12. Droplet Digital PCR (ddPCR)

QX200 Droplet Digital PCR and EvaGreen Digital PCR Supermix (Bio-Rad, #1864034) were used for ddPCR detection of the CUT&RUN enriched products. After diluting the samples, the reaction system was prepared according to the instructions provided with the kit. The prepared solution was transferred to a droplet generation card, and droplet generation oil (70 μL; Bio-Rad, #1864005) was added. Droplets were prepared using a droplet generator (Bio-Rad, #1864002) and transferred to a 96-well plate for PCR amplification, with the reaction program also following the instructions provided with the kit. After the PCR reaction was completed, the droplet reader (Bio-Rad, #1864003) and Bio-Rad QuantaSoft™ Analysis Pro software were used to read and analyze the positive droplets.

### 4.13. Super-Shift Electrophoretic Mobility Shift Assay (Super-Shift EMSA)

The extraction of nuclear proteins was performed using NE-PER Nuclear and Cytoplasmic Extraction Reagents (Thermo Fisher, 78833) following the instructions provided with the kit. The concentration of nuclear proteins was determined using the BCA method. Probes for the target gene’s promoter were designed and synthesized, including a biotin-labeled wild-type probe, a biotin-labeled mutant probe, and an unlabeled cold probe that is identical to the wild-type probe but not labeled. The LightShift Chemiluminescent EMSA Kit (Thermo Fisher, 20148) was used for EMSA analysis. The wild-type probe was incubated with 30 μg of nuclear protein, and in competition experiments, 200-fold excess of mutant and cold probes was added. In the super-shift experiment, a specific antibody against RORA was added. A 6.5% native polyacrylamide gel was used to separate the nucleic acid–protein complexes, with the reaction system and detailed steps following the instructions provided with the kit.

### 4.14. Statistical Analysis

Each experiment included at least three biological replicates, and a one-way analysis of variance and Student’s *t*-test were used to assess statistical significance. All statistical analyses were performed using GraphPad Prism 9.5.1 software, and the results were expressed as mean ± standard deviation; *p* < 0.05 was considered to indicate a statistically significant result.

## 5. Conclusions

In conclusion, our study reveals that the activation of RORA induces cell cycle arrest in HFSCs, subsequently suppressing their proliferation. Furthermore, the activation of RORA also promotes apoptosis in HFSCs, and the synergistic effects of these two processes lead to a notable decline in the cellular viability of HFSCs. Additionally, we found that RORA influences the expression of genes associated with ferroptosis, and we confirmed the binding interaction between RORA and the promoter regions of the cyclin genes *Ccna2* and *Ccne1*. Melatonin exhibits a remarkable stimulatory effect on the viability of HFSCs, potentially through RORA. This research could offer a theoretical foundation for studies on hair follicle development and seasonal molting in animals.

## Data Availability

The original contributions presented in this study are included in the article/Supplementary Materials. Further inquiries can be directed to the corresponding author.

## Supplementary Materials

The following supporting information can be downloaded at https://www.mdpi.com/article/10.3390/ijms26041579/s1.
