# Supplementary material for: The Retinoic-Acid-Related Orphan Receptor Alpha May Be Highly Involved in the Regulation of Seasonal Hair Molting
Source: Int J Mol Sci. 2025 Feb 13;26(4):1579. doi: 10.3390/ijms26041579 (PMC11855665; doi:10.3390/ijms26041579)
Supplement: Supplementary file 1 [file ijms-26-01579-s001.zip › File S1ú║Supplementary Materials.pdf]

Supplementary Materials for

The retinoic acid-related orphan receptor alpha may be highly involved in the regulation of seasonal hair molting

Yu Zhang *et al.*

*Corresponding author. Email: zwfur@nefu.edu.cn

This file includes:

Figure. S1 to S5

Table. S1 to S3

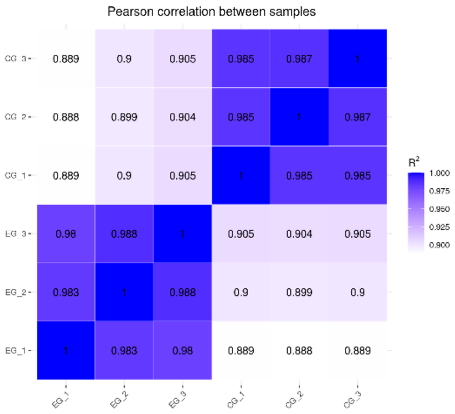

Figure S1. Pearson correlation coefficient is used to describe the reproducibility of biological replicates in RNA-seq results. The CG group was treated with 1000ng/L melatonin, while the EG group was treated with 1000ng/L melatonin in combination with SR1078.

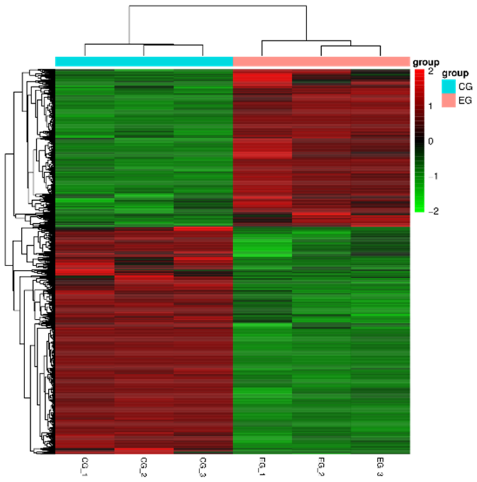

Figure S2. The results of clustering of significantly differentially expressed transcripts.

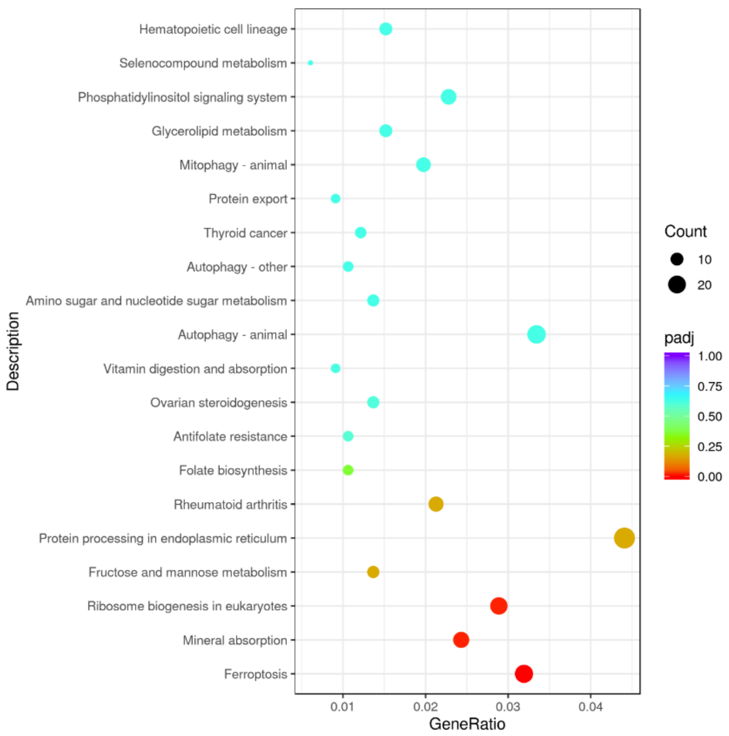

Figure S3. The KEGG enrichment analysis result of up-regulated transcripts in differentially expressed transcripts between EG group and CG group. The CG group was treated with 1000ng/L melatonin, while the EG group was treated with 1000ng/L melatonin in combination with SR1078.

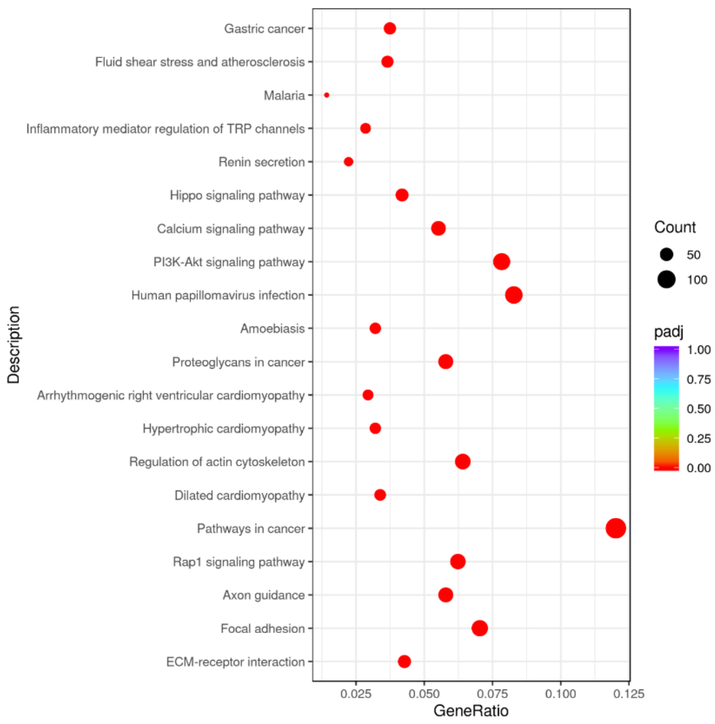

Figure S4. The KEGG enrichment analysis result of down-regulated transcripts in differentially expressed transcripts between EG group and CG group. The CG group was treated with 1000ng/L melatonin, while the EG group was treated with 1000ng/L melatonin in combination with SR1078.

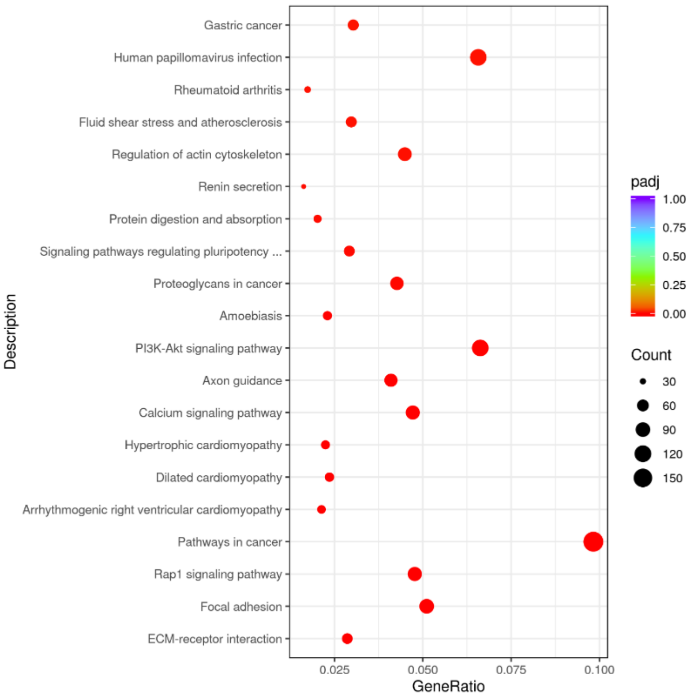

Figure S5. The KEGG enrichment analysis result of all differentially expressed transcripts between EG group and CG group. The CG group was treated with 1000ng/L melatonin, while the EG group was treated with 1000ng/L melatonin in combination with SR1078.

Table S1. Some differentially expressed genes in RNA-seq results (In the table, the values represent FPKM values).

Gene	EG_1	EG_2	EG_3	CG_1	CG_2	CG_3
Aifm2	5.15	7.42	7.66	11.26	13.10	13.39
Tfrc	3973.82	4035.32	4021.38	1270.53	1217.83	1188.18
Arf6	262.77	258.97	253.45	151.61	171.40	158.61
Gclc	101.47	78.05	72.48	36.41	32.18	35.31
Hspb1	58.45	62.32	76.06	163.52	158.53	168.10
Aco1	19.90	21.82	22.70	35.38	37.04	37.29
Atf4	801.39	682.25	654.93	335.53	291.42	288.71
Acsl4	37.15	31.58	29.24	19.22	17.22	16.60
Chac1	137.68	121.67	111.89	32.26	38.81	34.88
Keap1	36.31	31.00	28.70	20.36	22.65	20.69
Sqstm1	2188.87	2044.14	1941.95	202.23	189.04	201.70
Slc3a2	1817.72	1601.61	1409.46	214.46	220.37	209.55
Slc7a11	51.37	21.75	19.56	4.12	4.35	3.91
Tfrc	49.44	50.21	50.03	15.81	15.15	14.78
Sat1	241.98	234.30	229.10	39.25	32.94	32.96
Steap3	13.19	15.54	15.43	8.19	10.34	9.00
Hmox1	748.04	393.62	362.78	23.27	26.74	27.22
Acta2	50.46	43.48	40.07	5491.28	5557.09	5349.48
Actb	614.72	590.31	592.40	6752.00	6596.27	6773.23

Actg1	296.46	212.44	210.50	1894.36	1811.05	1914.20
Actg2	12.24	8.62	6.23	342.60	329.58	338.75
Tuba1a	83.06	84.49	88.77	453.30	437.08	437.48
Tuba1b	209.39	232.40	233.43	763.46	859.54	801.13
Tubb2a	3.76	5.56	4.75	15.65	15.70	14.66
Tubb4b	97.98	97.79	105.21	184.46	186.88	194.63
Tubb5	344.01	301.84	305.62	1320.89	1395.85	1378.49
Tubb6	84.93	78.14	81.33	288.56	312.58	303.69
Pcdh19	1.60	1.36	2.13	4.78	5.68	4.55
Itga1	1.89	2.66	2.70	13.91	13.91	13.19
Itga5	18.18	24.30	25.79	48.49	53.64	53.03
Itga6	1.01	1.46	0.92	19.69	19.55	19.78
Itgb1	255.26	267.07	277.36	718.08	659.43	681.03
Itgb5	4.60	5.78	6.86	36.22	38.29	36.62
Ppib	465.58	405.13	347.92	440.65	407.18	404.14
Gapdh	594.81	674.42	675.87	1243.73	1227.04	1234.61
Foxc1	669.96	895.23	810.87	3939.35	4189.80	4140.44
Lmna	185.53	177.05	173.04	400.60	424.18	412.15

Table S2. The primers used in this research

Primer Name	Sequence 5’-3’
PPIBF	GGCACAGGAGGAAAGAGCAT
PPIBR	ACCACATCCATGCCTTCCAG
GPX4F	GGAGGCAGGAGCCAGGAAGTAA
GPX4R	CACCACGCAGCCGTTCTTATCA
ACSL4F	AATGTCCGCATGATGCTGTCCG
ACSL4R	AACCACCTTCCTGCCAGTCCTT
TFRCF	TCGTGGAGACTACTTCCGTGCT
TFRCR	TGAGAGGGTGTGAGAGCCAGAG
CDK6F	CGTGACCTGAAGCCACAGAACA
CDK6R	GATGCAGCCAACACTCCAGAGG
BaxF	AGACACCTGAGCTGACCTTGGA
BaxR	TGCCACACGGAAGAAGACCTCT
Bcl2F	GGGAGCGTCAACAGGGAGATGT
Bcl2R	GCGTCTTCAGAGACAGCCAGGA
CCNA2F	GCCTGCCAACTGCAAGGTAGAA
CCNA2R	TGCTGTGGTGCTTTGAGGTAGG
CCNB1F	TGAGCCTGAGCCTGAACCTGTT
CCNB1R	TTGGATCACCACCATCGTCTGC
CCND2F	CGCTCTGTGTGCTACCGACTTC
CCND2R	TGTGCTGCTCTTGACGGAACTG
CCNE1F	GTGTCCTCGCTGCTTCTGCTT
CCNE1R	GCTGTTGGTGTGGGTCTGGATG
CCNE2F	GCCACCTGTACTGTCTGGAGGA

CCNE2R	CTCCTGTGAACATGCCCAGCTT
CDK1F	TGCAGAGCTGGCGACCAAGAA
CDK1R	CGAGAGCAAGTCCAAGCCGTTT
CDK2F	TCTGCCATTCTCACCGTGTCCT
CDK2R	CGGGTCACCATTTCGGCAAAGA
CDK8F	GCAGCAGCAGCAGGGCAATA
CDK8R	TGGAACGCTGATAGTCGGAGGT
CDK14F	ACGCCTTTCACAGCCATCAGAG
CDK14R	AGACAGCCCTCGCAGCAACT
CCNA2F	GCCTGCCAACTGCAAGGTAGAA
CCNA2R	TGCTGTGGTGCTTTGAGGTAGG
CCNB1F	TGAGCCTGAGCCTGAACCTGTT
CCNA2 for CUT F	GCTGACAGGAATGCTGACACTC
CCNA2 for CUT R	GCCTTCCGCAACTCTCCGAT
CCNE1 for CUT F	CAGGTGCATCACTGCGGTCAA
CCNE1 for CUT R	CGCCCGAAGGATTAGGAGCTTG

Table S3. The primary antibody used in this research

Protein	Information
RORα (for CUT&RUN)	Abcam ab256799
RORα (for EMSA)	Santa Cruz sc-518081
PPIB	Proteintech Group 11607-1-AP
CDK6	Proteintech Group 14052-1-AP
BCL2	Proteintech Group 68103-1-Ig
BAX	Proteintech Group 50599-2-Ig
SAT1	Proteintech Group 10708-1-AP
ACSL4	Proteintech Group 22401-1-AP
STEAP3	Proteintech Group 17186-1-AP
